# Genotypic Variation in Yield, Yield Components, Root Morphology and Architecture, in Soybean in Relation to Water and Phosphorus Supply

**DOI:** 10.3389/fpls.2017.01499

**Published:** 2017-08-29

**Authors:** Jin He, Yi Jin, Yan-Lei Du, Tao Wang, Neil C. Turner, Ru-Ping Yang, Kadambot H. M. Siddique, Feng-Min Li

**Affiliations:** ^1^College of Agriculture, Guizhou University Guiyang, China; ^2^State Key Laboratory of Grassland Agro-ecosystems, Institute of Arid Agroecology, School of Life Sciences, Lanzhou University Lanzhou, China; ^3^The UWA Institute of Agriculture and UWA School of Agriculture and Environment, The University of Western Australia, Perth WA, Australia; ^4^Dryland Agricultural Institute, Gansu Academy of Agricultural Sciences (GAAS) Lanzhou, China

**Keywords:** *Glycine max*, low P availability, root branching, root distribution, root length, water stress

## Abstract

Water shortage and low phosphorus (P) availability limit yields in soybean. Roots play important roles in water-limited and P-deficient environment, but the underlying mechanisms are largely unknown. In this study we determined the responses of four soybean [*Glycine max* (L.) Merr.] genotypes [Huandsedadou (HD), Bailudou (BLD), Jindou 21 (J21), and Zhonghuang 30 (ZH)] to three P levels [applied 0 (P0), 60 (P60), and 120 (P120) mg P kg^-1^ dry soil to the upper 0.4 m of the soil profile] and two water treatment [well-watered (WW) and water-stressed (WS)] with special reference to root morphology and architecture, we compared yield and its components, root morphology and root architecture to find out which variety and/or what kind of root architecture had high grain yield under P and drought stress. The results showed that water stress and low P, respectively, significantly reduced grain yield by 60 and 40%, daily water use by 66 and 31%, P accumulation by 40 and 80%, and N accumulation by 39 and 65%. The cultivar ZH with the lowest daily water use had the highest grain yield at P60 and P120 under drought. Increased root length was positively associated with N and P accumulation in both the WW and WS treatments, but not with grain yield under water and P deficits. However, in the WS treatment, high adventitious and lateral root densities were associated with high N and P uptake per unit root length which in turn was significantly and positively associated with grain yield. Our results suggest that (1) genetic variation of grain yield, daily water use, P and N accumulation, and root morphology and architecture were observed among the soybean cultivars and ZH had the best yield performance under P and water limited conditions; (2) water has a major influence on nutrient uptake and grain yield, while additional P supply can modestly increase yields under drought in some soybean genotypes; (3) while conserved water use plays an important role in grain yield under drought, root traits also contribute to high nutrient uptake efficiency and benefit yield under drought.

## Introduction

Soybean [*Glycine max* (L.) Merr.] is one of the 10 most widely grown crops. Previous studies have shown that drought stress led to a 24 to 50% reduction in seed yield (Frederick et al., 2001; Sadeghipour and Abbasi, 2012) and P deficit significantly reduced soybean yield (Xu et al., 2003; Jin et al., 2006). Drought (Manavalan et al., 2009) and low phosphorus (P) availability (Schachtman et al., 1998; Xu et al., 2003) are two important factors that limit its yield and yield stability. Phosphorus is a scarce and non-renewable resource (Cordell et al., 2009; Gilbert, 2009) and drought incidents (both intensity and frequency) are predicted to increase with climate change (Mpelasoka et al., 2008; Turner et al., 2011). Drought could restrict the soil P diffusion and P uptake in plants (Suriyagoda et al., 2014). If yields are to be maintained when water and P availability are limited, then it is important to understand plant performance and adaptation to moisture- and P-limitations.

Phosphorus (P) is essential to plants. P deficiency reduces the P concentration in leaves and the critical P concentrations are different in different species (Pinkerton et al., 1997). P deficiency restricts plant growth by reducing photosynthetic rate and stomatal conductance (Jacob and Lawlor, 1991; Ghannoum and Conroy, 2007). Phosphorus fertilization is the most efficiency way to increase P availability; applied P increases water use efficiency (Payne et al., 1992), drought tolerance (Singh and Sale, 2000; Garg et al., 2004; Jones et al., 2005) and shoot dry matter (Rodriguez et al., 1996) in many plant species under drought stress, while adequate soil P levels have been shown to offset the impact of drought on the yield and quality of malting barley (Jones et al., 2003). In soybean, applied P alleviated the negative effect of drought stress on yield (Jin et al., 2006), but the underlying mechanisms are not known. Moreover, the responses of yield and its components in both water-limited and P-deficient environments have not been studied in soybean.

Phosphorus acquisition by plants in most environments is restricted by low mobility and availability of P in soil (Schachtman et al., 1998) and by soil drying (Suriyagoda et al., 2010, 2014). Maximizing the ability of the root to absorb P from the soil is one of the main mechanisms to cope with the P deficiency. Higher P acquisition by plants depends on root morphology (Raghothama, 1999, 2000) and architecture (Lynch, 1995; Lynch and Brown, 2001) such as: (1) greater root branching, (2) greater root length density and a higher fraction of roots in surface soil layers, (3) greater production of thin roots, and (4) partitioning of more plant biomass to the root system (Lynch and Brown, 2001; White et al., 2005; Lynch, 2007, 2011; White and Hammond, 2008; Richardson et al., 2011). Thus, altering the morphology and architecture of roots is a powerful way for crop plants to maximize root absorption and acquisition of P. These adaptations, however, may be at the expense of the acquisition of deeper soil resources, such as water (Lynch, 2007; Suriyagoda et al., 2010), if the density of shallow roots increases. Previous studies have shown that increasing root length density in deep soil improves the yield under drought by increasing water uptake (Henry et al., 2011; Lilley and Kirkegaard, 2011). Furthermore, higher lateral root densities (LRDs) may increase competition for nitrogen (N) between lateral roots, and reduce N uptake per unit root length (Postma et al., 2014), but how root morphology and architecture contribute to P uptake under water and P deficits is not known.

The objective of this study was to investigate water- and P-acquisition strategies in soybean, with special reference to root morphology and root architecture, under both water-limited and P-deficient conditions and adequately watered and P-sufficient conditions. In a previous study, we observed considerable variation in grain yield and root length density among eight soybean genotypes under well-watered and drought conditions (He et al., 2017), Four of these eight genotypes — Huandsedadou, Bailudou, Jindou 21, and Zhonghuang 30 — with varying grain yields and root length densities under drought conditions were selected to compare biomass accumulation and allocation, N and P accumulation, P and N uptake per unit root length, root morphology and architecture, and yield and its components when grown under both water-limited and P-deficient and well-watered and P-sufficient conditions. Our hypothesis was that genotypes with greater root length and branching would have greater N and P uptake and higher yields in low P and water-limited environments than genotypes with smaller root lengths and less branching.

## Materials and Methods

### Materials and Growth Conditions

A three-factorial randomized complete block design (genotypes, soil P applications, and water treatments) experiment was conducted from 29 April to 3 October 2015 at the Yuzhong Experiment Station (35° 51′ N, 104° 07′ E, altitude 1,620 m) of Lanzhou University in Yuzhong County, Gansu Province, China. Four soybean genotypes — Huandsedadou (HD), Bailudou (BLD), Jindou 21 (J21), and Zhonghuang 30 (ZH) — that had different yields and water use in pot experiments conducted in 2014 (He et al., 2017) were selected. The genotypes were grown in pots in an open rainout shelter that could be closed when rain threatened. A total of 144 long cylindrical pots (1.05 m long and 0.16 m diameter) were used and filled with 18.6 kg of a sieved, loess soil-based substrate [loess soil:vermiculite (v:v) = 3:1]. The soil depth was 1.0 m. The loess soil, obtained from a field at the experiment station, has a silty-loam texture, similar to an Entisol (United States Soil Conservation Service 1975). Transparent polyethylene sleeves (1.3 m long, 0.24 m wide, 101 μm thick) were placed inside the pots before filling for ease of removal of the soil and roots at harvest. The initial concentration of available P in the soil:vermiculite mixture was about 2 mg kg^-1^. Three P levels: 0 (P0), 60 (P60), and 120 (P120) mg P kg^-1^ dry soil were applied to the top 0.4 m of soil (to mimic the P distribution in the field). Phosphorus was added as ammonium dihydrogen phosphate (NH_4_H_2_PO_4_) that was ground to a powder and mixed with the soil mixture in an end-over-end shaker.

Each cylinder was weighed and watered to maintain the soil water content (SWC) at about 100% field capacity (FC), which was determined by watering the soil until it became free draining and then allowing the water to drain for 24 h before weighing. Seeds were initially placed in water containing 5 g L^-1^ carbendazim for 600 s to prevent disease; on 29 April 2015 two seeds were sown in each cylinder and thinned to one after germination. Black plastic film was placed on the top of the cylinders to prevent water loss by soil evaporation. Two water treatments were then imposed: (i) well-watered (WW) in which SWC was maintained between 85 and 100% FC, and (ii) a drought cycle (WS) in which water was withheld until the SWC decreased to 30% FC and then rewatered to 100% FC. The drought–rewater cycle was repeated until maturity and required rewatering 3–5 times.

### Water Use Determination

Plants in the WW treatment were weighed and watered every 4 days to maintain the soil between 80 and 100% FC until maturity [136–147 days after sowing (DAS)]. In the WS treatment, cylinders were weighed every 4–5 days until maturity and rewatered to 100% FC when the SWC reached 30% FC. During the drying cycle, the amount of water used was determined by the reduction in pot weight. In the WW treatment, water use was determined from the water added. There were three replicates (pots) per genotype per treatment. Half of the pots (4 genotypes × 3 P levels × 2 water treatments × 3 replicates = 72 pots) were harvested at 65 DAS and half were harvested at maturity.

### Sampling at 65 Days after Sowing

At 65 DAS, the shoots in half of the pots were cut ∼10 mm from the soil surface, dried in an oven at 80°C for 48 h, weighed and then stored for total P and total N analysis. The polyethylene sleeves were removed from the pots and divided into three sections [upper (0–0.4 m), middle (0.4–0.6 m), and lower (0.6–1.0 m)] to determine the root distribution in the different soil layers. The soil was carefully washed from the roots over a 0.2-mm sieve. Root density was determined for lateral and adventitious roots according to Fenta et al. (2014) with some modifications. Adventitious roots were counted and their root length measured. The lateral roots were counted within a 50-mm segment from where the lateral root emerged. A 50-mm root segment from the base of three randomly selected adventitious and lateral roots was selected to count rootlet numbers to determine adventitious and lateral root branching density. Root density = root number/length (50 mm) of root used to determine root number.

After determining root density, the root length of each part was determined by scanning with an Epson 10000XL (Epson, Inc., Long Beach, CA, United States) scanner and analyzing the root samples using WinRHIZO Pro (Régent Instruments, Inc., Quebec City, QC, Canada). After scanning, roots were dried at 80°C for 48 h, weighed, then stored for P and N analysis. Root-to-shoot ratio = root dry weight/shoot dry weight.

### Determination of P and N Concentration

Samples were ground to a fine powder with an Ultra Centrifugal Mill (ZM200, Retsch, GmbH, Düsseldorf, Germany). Approximately 150 mg subsamples were digested with H_2_O_2_–H_2_SO_4_. Total N and P concentrations in the plant tissues were determined using the Kjeldahl method (SKD-800, Shanghai Peiou Analytical Instruments, Co. Ltd., Shanghai, China) and the molybdenum–stibium anti-spectrophotometry method (UV-1800 Spectrophotometer, Shanghai Meipuda Instrument, Co. Ltd., Shanghai, China), respectively (Ren et al., 2011). The sum of the P and N concentrations multiplied by the dry weight (DW) of shoots and roots represented P and N accumulation in the shoots and roots, respectively. P and N uptake per root length = P and N accumulation in the whole plant/total root length (Aziz et al., 2017).

### Final Harvest

Whole plants (4 genotypes × 3 P levels × 2 water treatments × 3 replicates = 72 pots) were harvested at physiological maturity, defined as when 95% of the pods were brown (Fehr et al., 1971). The filled pod number (we defined the pod with seeds as filled pod), grain number and yield were determined. The roots were washed from the soil; root dry weight (RDW) and density were measured as described above at 65 DAS. Water use for the whole lifecycle was calculated by adding up the water use from sowing to physiological maturity. The following variables were calculated: water use efficiency for grain yield (WUE_G,_ g L^-1^) = grain yield/water use; 100-grain weight (HGW, g) = grain yield/grain number × 100; and mean daily water use (mL plant^-1^) = water use over the whole lifecycle/days from sowing to maturity.

### Statistics

The experiment was a three-factorial randomized complete block design (genotype, soil P application, and water treatment). All measured variables were analyzed by general analysis of variance (ANOVA) using the GenStat 17.0 statistical package (VSN International, Ltd., Rothamsted, England). Mean comparisons were made by LSD at *P* = 0.05 significance level. The data used in the figures are means + one standard error of the mean of three replicates.

## Results

### Shoot and Root Dry Matter Accumulation, Root-to-Shoot Ratio, P and N Accumulation, and P and N Uptake Efficiency at 65 DAS

**FIGURE 1:**
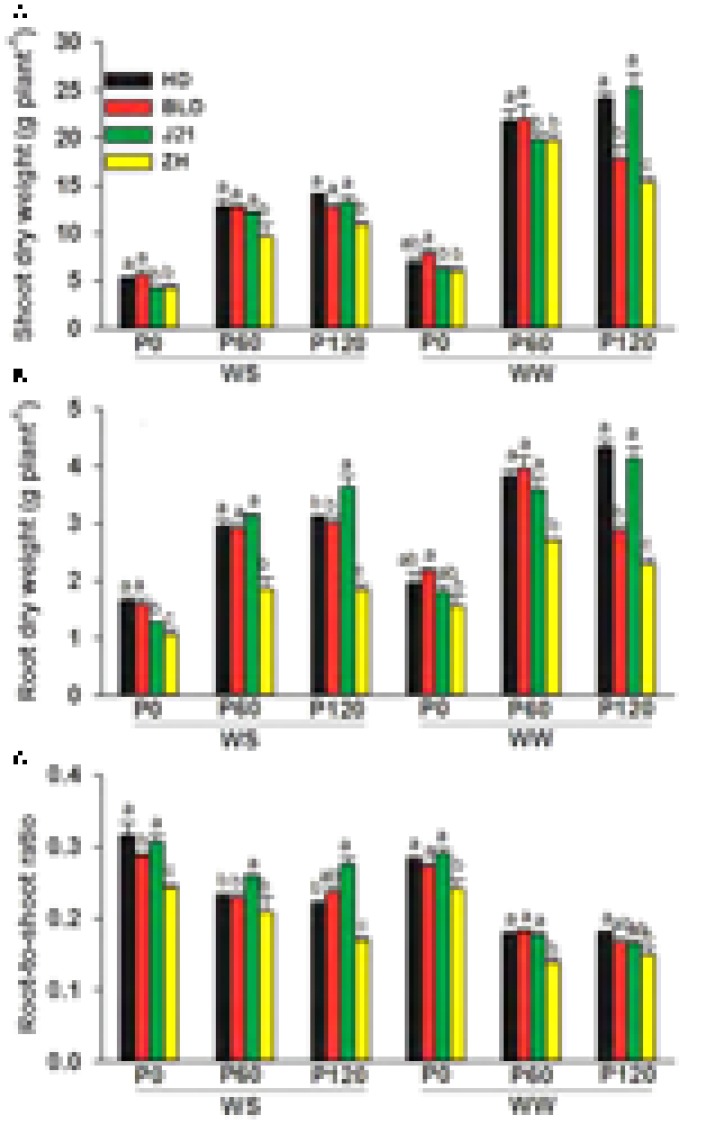
**(A)** Shoot dry weight, **(B)** root dry weight, and **(C)** root-to-shoot ratio of four soybean genotypes [Huangsedadou (HD), Bailudou (BLD), Jindou 21 (J21), and Zhonghuang 30 (ZH)] under two water treatments [well-watered (WW) and cycles of water stress (WS)] and three P levels [0 (P0), 60 (P60), and 120 (P120) mg P kg^-1^ dry soil] at 65 days after sowing. Values are means + one standard error of the mean (*n* = 3). Means with different letters are significantly different at *P* = 0.05.

Averaging across genotypes and water treatments, shoot and root dry weights increased by 184 and 85%, respectively, and the root-to-shoot ratio (R:S) decreased by 41% with applied P [values are the average of P60 and P120 which did not differ significantly (**Figure 1** and Supplementary Table S1)]. Averaging across genotypes and applied P treatments, water stress decreased shoot and root DWs by 39 and 21%, respectively, and R:S increased by 13% (**Figure 1** and Supplementary Table S1), indicating that more dry matter was partitioned to roots under water stress. The shoot and root DWs varied between genotypes; ZH had significantly lower shoot DW than the other three genotypes (**Figure 1**) at P60 and P120 in the WS treatment and P120 in the WW treatment. ZH also had significantly (*P* < 0.05) lower root DW (RDW) than the other genotypes at all three P levels and both water treatments. Averaged across genotypes, shoot DW, but not root DW, decreased more in the WS treatment at high P (P60 and P120) than when P was deficient (P0) (**Figure 1** and Supplementary Table S1), such that R:S was unchanged at P0 by water stress, but decreased at P60 and P120 in the WW treatment (**Figure 1**).

**FIGURE 2:**
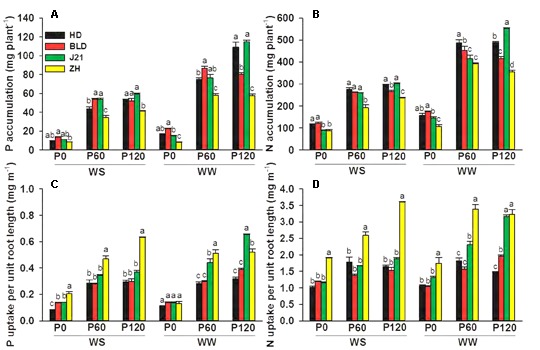
**(A)** P accumulation, **(B)** N accumulation, **(C)** P uptake per unit root length, and **(D)** N uptake per unit root length of four soybean genotypes [Huangsedadou (HD), Bailudou (BLD), Jindou 21 (J21), and Zhonghuang 30 (ZH)] under two water treatments [well-watered (WW) and cycles of water stress (WS)] and three P levels [0 (P0), 60 (P60), and 120 (P120) mg P kg^-1^ dry soil] at 65 days after sowing. Values are means + one standard error of the mean (*n* = 3). Means with different letters are significantly different at *P* = 0.05.

Averaging across genotypes and applied P treatments, water stress significantly reduced both P and N accumulation by 40%, but had no effect on P uptake per unit root length and only had a small effect on N uptake per unit root length (**Figure 2** and Supplementary Table S1). Averaging across genotypes and water treatments, increased P supply increased P accumulation by 363% at P60 and 446% at P120, N accumulation by 174% at P60 and 192% at P120, P uptake per unit root length by 169% at P60 and 223% at P120, and N uptake per unit root length by 57% at P60 and 76% at P120. ZH had significantly lower P accumulation, particularly at high P supply, but significantly higher P uptake per unit root length (except P120 under WW) (**Figure 2** and Supplementary Table S1). In line with shoot DW, P and N accumulation, but not P and N uptake per unit root length, decreased more with water stress at high P than when P was deficient (P0) (**Figure 2** and Supplementary Table S1).

### Root Morphology, Distribution, and Architecture at 65 DAS and Maturity

**FIGURE 3:**
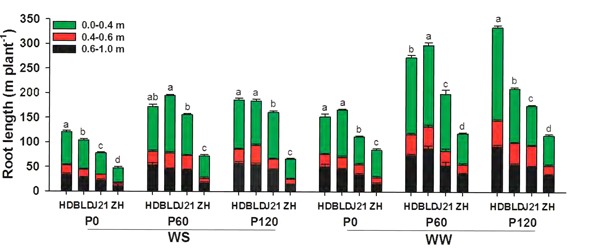
Root length (m plant^-1^) in the upper (0–0.4 m), middle (0.4–0.6 m), and bottom (0.6–1.0 m) of the soil profile in four soybean genotypes [Huangsedadou (HD), Bailudou (BLD), Jindou 21 (J21), and Zhonghuang 30 (ZH)] under two water treatments [well-watered (WW) and cycles of water stress (WS)] and three P levels [0 (P0), 60 (P60), and 120 (P120) mg P kg^-1^ dry soil] at 65 days after sowing. Values are means + one standard error of the mean (*n* = 3). Means with different letters are significantly different at *P* = 0.05.

**FIGURE 4:**
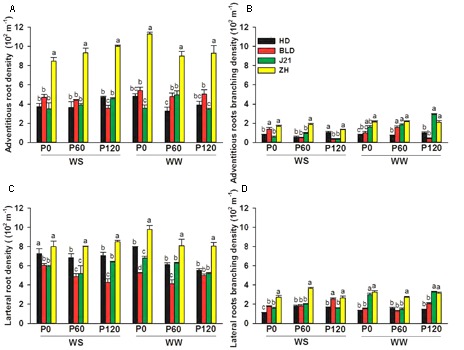
**(A)** Adventitious root density (10^2^ m^-1^), **(B)** adventitious root branching density (10^2^ m^-1^), **(C)** lateral root density (10^2^ m^-1^), and **(D)** lateral root branching density (10^2^ m^-1^) of four soybean genotypes [Huangsedadou (HD), Bailudou (BLD), Jindou 21 (J21), and Zhonghuang 30 (ZH)] under two water treatments [well-watered (WW) and cycles of water stress (WS)] and three P levels [0 (P0), 60 (P60), and 120 (P120) mg P kg^-1^ dry soil] at 65 days after sowing. Values are means + one standard error of the mean (*n* = 3). Means with different letters are significantly different at *P* = 0.05.

Averaging across genotypes and applied P treatments, limited water significantly reduced total root length in the 0–1.0 m soil layer by 31%, decreased adventitious root branching density by 62%, but had no effect on adventitious root density (ARD), LRD, or lateral root branching density (**Figures 3, 4** and Supplementary Table S2). Averaging across genotypes and water treatments, applied P significantly increased total root length by 72% at P60 and P120. Applied P reduced LRD by 12% at P60 and P120, but had no effect on ARD, adventitious root branching density or lateral root branching density (**Figures 3, 4** and Supplementary Table S2). Compared with the other three genotypes, ZH had significantly shorter total root length in all treatments (**Figure 3**), significantly higher ARD and LRD, higher adventitious and lateral root branching densities (**Figure 4**), but lower root length in the 0–0.4 m soil layer, particularly in the WS treatment and at P0 in the WW treatment (**Figure 3**).

At maturity, RDW was about 10-fold greater than at 65 DAS (RDW_M_ = 10.4RDW_65_ + 5.3, *R*^2^= 0.5, *P* < 0.001), while LRD and ARD at maturity were both about 90% of the values at 65 DAS (LRD_M_ = 0.9LRD_65_ + 1.34, *R*^2^= 0.7, *P* < 0.001; ARD_M_ = 0.9ARD_65_ + 1.07, *R*^2^= 0.7, *P* < 0.001) (data not shown).

### Yield Performance and Yield Components

**FIGURE 5:**
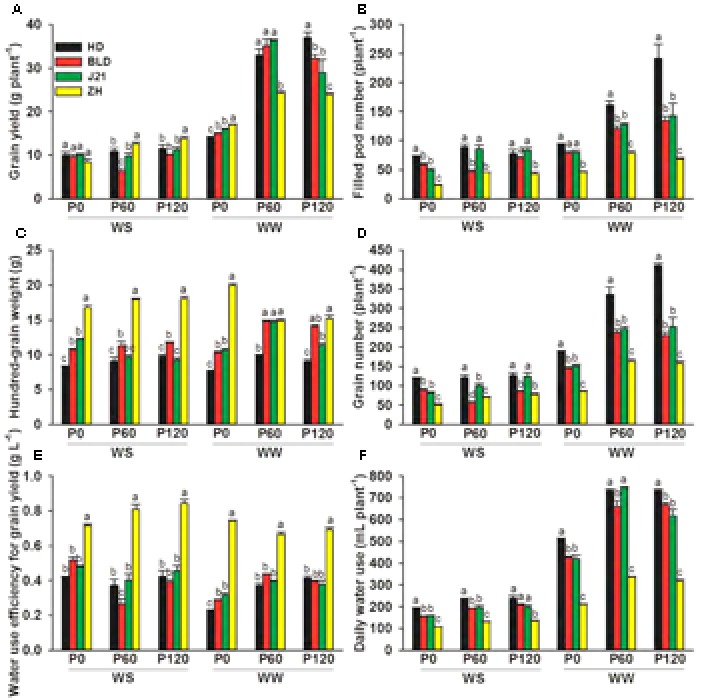
**(A)** Grain yield (g plant^-1^), **(B)** filled pod number (plant^-1^), **(C)** 100-grain weight (g), **(D)** grain number (plant^-1^), **(E)** water use efficiency for grain yield (g L^-1^), and **(F)** water use (mL plant^-1^ day^-1^) of four soybean genotypes [Huangsedadou (HD), Bailudou (BLD), Jindou 21 (J21), and Zhonghuang 30 (ZH)] under two water treatments [well-watered (WW) and cycles of water stress (WS)] and three P levels [0 (P0), 60 (P60), and 120 (P120) mg P kg^-1^ dry soil] at maturity. Values are means + one standard error of the mean (*n* = 3). Means with different letters are significantly different at *P* = 0.05.

Averaging across genotypes and applied P treatments, water stress significantly reduced grain yield by 60%, filled pod number by 46%, grain number by 58%, daily water use by 66% and 100-grain weight from 12.7 to 12.0, but increased water use efficiency for grain yield from 0.44 to 0.51 g L^-1^ (**Figure 5** and Supplementary Table S3). Averaging across genotypes and water treatments, increased P supply increased grain yield by 68%, and filled pod number by 61% at P60 and P120 (values at P60 and P120 did not differ significantly), grain number by 46% at P60 and 61% at P120, and daily water use by 48% at P60 and 43% at P120, but had little effect on 100-grain weight and no effect on water use efficiency for grain yield (**Figure 5** and Supplementary Table S3), indicating that P supply increased grain yield mainly by increasing grain number. Grain yield, filled pod number, grain number and daily water use decreased more with water stress at high P than when P was deficient (P0) (**Figure 5** and Supplementary Table S3). Genotypic differences highlight the interaction between water supply and P supply; ZH had significantly higher grain yield at P60 and P120 in the WS treatment and at P0 in the WW treatment than the other three genotypes (**Figure 5A**). In the WS treatment, applied P increased grain yield in HD and ZH by 9%, and 51%, respectively, but reduced yields in BLD and J21 by 34 and 2% at P60, respectively. In the WS treatment at P120, grain yield increased in HD, BLD, J21, and ZH by 25, 6, 14, and 65%, respectively, compared with P0.

### Association between Root Characteristics, P and N Accumulation, P and N Uptake per Unit Root Length, and Grain Yield

**FIGURE 6:**
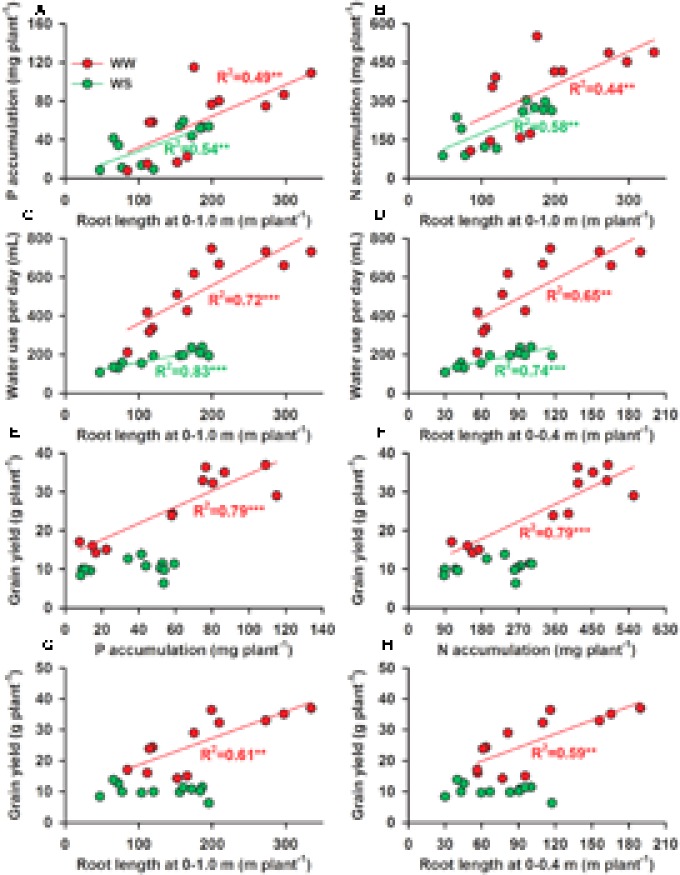
The relationships between **(A)** P accumulation (mg plant^-1^) and root length (m) at 0–1.0 m soil profile, **(B)** N accumulation (mg plant^-1^) and root length (m) at 0–1.0 m soil profile, **(C)** water use per day (mL plant^-1^) and root length at 0–1.0 m soil profile (m), **(D)** water use per day (mL plant^-1^) and root length at 0 to –0.4 m soil profile, **(E)** grain yield (g plant^-1^) and P accumulation (mg plant^-1^), **(F)** grain yield and N accumulation (mg plant^-1^), **(G)** grain yield and root length (m) at 0–1.0 m soil profile, **(H)** grain yield and root length (m) at 0–0.4 m soil profile in four soybean genotypes under two water treatments [well-watered (WW) and cycles of water stress (WS)] and three P levels [0 (P0), 60 (P60), and 120 (P120) mg P kg^-1^ dry soil]. The significant linear regression equations are shown, ^∗^*P* < 0.05, ^∗∗^*P* < 0.01, and ^∗∗∗^*P* < 0.001.

**FIGURE 7:**
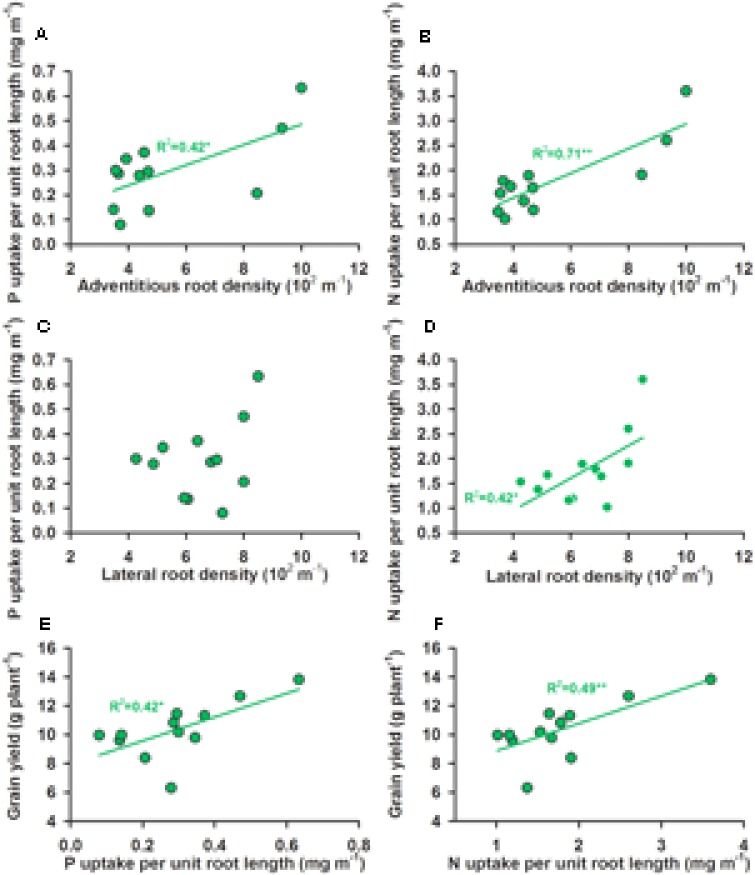
The relationships between **(A)** P uptake per unit root length (mg m^-1^) and adventitious root density (10^2^ m^-1^), **(B)** N uptake per unit root length (mg m^-1^) and adventitious root density (10^2^ m^-1^), **(C)** P uptake per unit root length (mg m^-1^) and lateral root density (10^2^ m^-1^), **(D)** N uptake per unit root length (mg m^-1^) and lateral root density (10^2^ m^-1^), **(E)** grain yield (g plant^-1^) and P uptake per unit root length (mg m^-1^) and **(F)** grain yield (g plant^-1^) and N uptake per unit root length (mg m^-1^) in four soybean genotypes given cycles of water stress (WS) and three P levels [0 (P0), 60 (P60), and 120 (P120) mg P kg^-1^ dry soil]. The significant linear regression equations are shown,^∗^*P* < 0.05, ^∗∗^*P* < 0.01, and ^∗∗∗^*P* < 0.001.

P and N accumulation were both positively correlated with root length over the whole soil profile and also in the 0–0.4 m soil layer (data not shown) in both the WW and WS treatments (**Figures 6A,B**). Water use per day was positively correlated with root length over the whole soil profile (0–1.0 m) (**Figure 6C**) and root length over the 0–0.4 m soil layer (**Figure 6D**) in both the WW and WS treatments, but water use was much less per unit root length in the WS treatment than the WW treatment (**Figures 6C,D**). In the WW treatment grain yield was positively and significantly correlated with P and N accumulation (**Figures 6E,F**) and with root length over the whole soil profile (**Figure 6G**) and also in the 0–0.4 m soil layer (**Figure 6H**), but not in the WS treatment (**Figures 6G,H**). In the WS treatment, but not in the WW treatment, P and N uptake per unit root length was positively and significantly correlated with ARD (**Figures 7A,B**) and N uptake was positively associated with LRD (**Figure 7C**). In the WS treatment, but not in the WW treatment, the P and N uptake per unit root length was positively and significantly associated with the increase in grain yield (**Figures 7E,F**).

## Discussion

This study showed that limited water and low P supply significantly reduced shoot and root dry weight and grain yield, these results being consistent with a previous study in soybean (Jin et al., 2006). The interaction between P supply and water availability showed that water stress reduced yields more at high P supply than at zero added P. Further, genotype also influenced the interaction, with ZH and to a lesser extent HD, having a higher grain yield than the other two genotypes with P supplied at 60 and 120 mg P kg^-1^ in the WS treatment and ZH having a higher grain yield than the other three genotypes with no added P in the WW treatment (**Figure 5**). ZH was chosen for this study because in a previous study it yielded as well as or higher than other genotypes under dryland field conditions and under water stress in a pot study in the glasshouse (He et al., 2017). In the present study, ZH had the least daily water use and greatest water use efficiency for grain yield than the other three genotypes at all three levels of P supply and both water treatments (**Figure 5**). This was consistent with our previous observation that conserved water use improved soybean yield performance under drought (He et al., 2017). At all levels of P and water supply, the least daily water use was observed in ZH among the four genotypes, and was associated with the smallest root DW and the shortest root length even though the roots extended throughout the 1-m soil profile (**Figures 1, 5**). The smaller water use resulted in only three rewatering events, compared to four or five in the other three genotypes, and presumably resulted in higher average SWCs over the drying and rewatering period. However, conserved water does not fully account for the higher yields in ZH in the WS treatment when P was added and at zero P in the WW treatment.

Nitrogen and P accumulation were significantly and positively correlated with grain yield under WW, but not under WS conditions. This was similar to observations in the previous study (Jin et al., 2006), in which the increase in grain yield by applied P under WW conditions was mainly caused by increasing P and N accumulation which was associated with the increase in root length by applied P in the WW treatment (**Figure 6**). The increase in N accumulation by P addition in shoots was indirect evidence of enhancement of N accumulation under drought stress, because P can improve N metabolism and increase soluble protein under drought (Al-Karaki et al., 1996). Our results also showed that grain yield was not significantly associated with P or N accumulation in the WS treatment even though N and P accumulation was increased by the increase of root length under WS (**Figure 6**). Moreover, we found that the grain yield in the WS treatment was associated with P and N uptake per unit root length (**Figure 7**), suggesting a role for P and N uptake per unit root length in the yield performance of soybean in dry soil.

Root morphology and root architecture are recognized as important in drought tolerance (Fenta et al., 2014) and the adaptation to low P availability (Zhao et al., 2004; Ao et al., 2010; Vandamme et al., 2013). In a previous study, He et al. (2017) showed that cultivars with a high yield had a lower root length than those with a low yield in the WS treatment, but the relationship between root morphology and architecture, water uptake, nutrient uptake and grain yield were not explored. Jin et al. (2005) suggested that P application could improve soybean grain yield under water stress by improving root morphology, but how the roots contributed to yield was not shown. In the present study, N and P accumulation were positively associated to the same degree with root length, and water use per day or over the whole season was positively associated with root length, but the uptake per unit root length in the WS treatment was less than in the WW treatment and grain yield was not associated with either root length or P and N accumulation in the WS treatment, unlike in the WW treatment (**Figure 7**). This suggests that overall root length was not a factor in determining yield in the WS treatment. However, under water-limited conditions, P and N uptake per unit root length was significantly and positively associated with grain yield, the density of adventitious roots was significantly and positively associated with N and P uptake per unit root length, and LRD was significantly and positively associated with N uptake per unit root length (**Figure 7**). These results indicate that high ARD improved P and N uptake efficiency (N and P uptake per unit root length), presumably through increased uptake area per unit soil volume with benefits for uptake of immobile nutrients such as P from dry or intermittently wet soil. Thus, root morphology and root architecture had benefits for the uptake of water and nutrients and, in turn, on yield under water and P deficits.

In summary, genetic variation of grain yield, daily water use, P and N accumulation, and root morphology and architecture were observed among the soybean genotypes and ZH with higher ARD and LRD had the best yield performance under P and water limited conditions. Grain yield, shoot and root DWs, daily water use, and P and N accumulation significantly decreased with water stress. Applied P significantly increased grain yield, shoot DW, daily water use, and P and N accumulation, but there was a significant interaction between water availability and applied P with a greater reduction in grain yield, shoot DW, daily water use, and P and N accumulation at high P than when P was deficient (P0). Under WW conditions, soybean grain yield was positively associated with root length, but not under intermittent drought conditions. However, in the WS treatment, our results suggested improved P and N uptake efficiency was associated with more adventitious and lateral root numbers per unit length of root, and this was associated with increased yield performance.

## Author Contributions

JH, YJ, NT, and F-ML conceived and designed the experiments. JH, YJ, and TW conducted experiments. Y-LD, R-PY, and KS contributed to the analysis and interpretation of the data. JH, YJ, NT, and F-ML wrote the manuscript; all authors contributed to the discussion and approved the final manuscript.

## Conflict of Interest Statement

The authors declare that the research was conducted in the absence of any commercial or financial relationships that could be construed as a potential conflict of interest.
