# Supplementary material for: Genotypic Variation in Yield, Yield Components, Root Morphology and Architecture, in Soybean in Relation to Water and Phosphorus Supply
Source: Front Plant Sci. 2017 Aug 29;8:1499. doi: 10.3389/fpls.2017.01499 (PMC5583600; doi:10.3389/fpls.2017.01499)
Supplement: Supplementary file 1 [file Table_1.DOCX]

**Supplementary data**

Table S1. Significance of genotype (G), water treatment (W), P level (P) and their interactions on shoot and root dry weight (DW) (g plant^-1^), root-to-shoot ratio (R:S), P and N accumulation (mg plant^-1^) and P and N uptake per unit root length (mg m^-1^) of four soybean genotypes [Huangsedadou (HD), Bailudou (BLD), Jindou 21 (J21) and Zhonghuang 30 (ZH)] under two water treatments (well-watered and cycles of water stress) and three P levels (0 , 60 and 120 mg P kg^-1^ dry soil). n.s. not significant, **P*<0.05, ***P*<0.01 and ****P*<0.001. The values in parenthesis are the LSD at *P*=0.05.

| Source of variability | Shoot DW | Root DW | R:S | P accumulation | N accumulation | P uptake per root length | N uptake per root length |
| --- | --- | --- | --- | --- | --- | --- | --- |
| G | *(2.0) | ***(0.40) | ***(0.021) | ***(6.7) | **(31) | ***(0.05) | ***(0.34) |
| W | ***(1.4) | ***(0.29) | ***(0.012) | ***(7.8) | ***(19) | n.s | *(0.24) |
| P | ***(1.7) | ***(0.34) | ***(0.021) | ***(5.6) | ***(2) | ***(0.04) | ***(0.30) |
| GxW | n.s | n.s | n.s | n.s | n.s | *(0.07) | n.s |
| GxP | n.s | *(0.69) | n.s | **(13.6) | n.s | *(0.08) | n.s |
| WxP | ***(2.5) | n.s | **(0.021) | ***(9.6) | ***(40) | n.s | n.s |
| GxWxP | n.s | n.s | n.s | n.s | n.s | n.s | n.s |
