# Supplementary material for: Genotypic Variation in Yield, Yield Components, Root Morphology and Architecture, in Soybean in Relation to Water and Phosphorus Supply
Source: Front Plant Sci. 2017 Aug 29;8:1499. doi: 10.3389/fpls.2017.01499 (PMC5583600; doi:10.3389/fpls.2017.01499)
Supplement: Supplementary file 2 [file Table_2.DOCX]

**Supplementary data**

Table. S2. Significance of genotype (G), water treatment (W), P level (P) and their interactions on root length at 0–1.0 m (m plant^-1^), root length at 0–0.4 m (m plant^-1^), adventitious root density (10^2^ m^-1^), adventitious root branching density (10^2^ m^-1^), lateral root density (10^2^ m^-1^) and lateral root branching density (10^2^ m^-1^) of four soybean genotypes [Huangsedadou (HD), Bailudou (BLD), Jindou 21 (J21) and Zhonghuang 30 (ZH)] under two water treatments (well-watered and cycles of water stress) and three P levels (0, 60 and 120 mg P kg^-1^ dry soil). n.s. not significant, **P*<0.05, ***P*<0.01 and ****P*<0.001. The values in parenthesis are the LSD at *P*=0.05.

| Source of variability | Root length at  0–1.0 m | Root length at  0–0.4 m | Adventitious root density | Adventitious root branching density | Lateral root density | Lateral root branching density |
| --- | --- | --- | --- | --- | --- | --- |
| G | ***(27) | ***(14) | ***(0.87) | ***(0.30) | ***(0.52) | ***(0.29) |
| W | ***(19) | ***(11) | n.s | ***(0.21) | n.s | n.s |
| P | ***(24) | ***(13) | n.s | n.s | ***(0.49) | n.s |
| GxW | n.s | *(21) | n.s | ***(0.43) | n.s | ***(0.41) |
| GxP | *(47) | **(27) | n.s | *(0.52) | n.s | *(0.50) |
| WxP | n.s | n.s | n.s | n.s | *(0.61) | ***(0.35) |
| GxWxP | n.s | n.s | n.s | *(0.74) | *(1.32) | *(0.71) |
