# Supplementary material for: Genotypic Variation in Yield, Yield Components, Root Morphology and Architecture, in Soybean in Relation to Water and Phosphorus Supply
Source: Front Plant Sci. 2017 Aug 29;8:1499. doi: 10.3389/fpls.2017.01499 (PMC5583600; doi:10.3389/fpls.2017.01499)
Supplement: Supplementary file 3 [file Table_3.DOCX]

**Supplementary data**

Table. S3. Significance of genotype (G), water treatment (W), P level (P) and their interactions on grain yield (g plant^-1^), filled pod number (plant^-1^), hundred-grain weight (g), grain number (plant^-1^), water use efficiency for grain yield (WUE, g L^-1^), daily water use (mL day^-1^ plant^-1^) of four soybean genotypes [Huangsedadou (HD), Bailudou (BLD), Jindou 21 (J21) and Zhonghuang 30 (ZH)] under two water treatments (well-watered and cycles of water stress ) and three P levels (0 , 60 and 120 mg P kg^-1^ dry soil). n.s. not significant, **P*<0.05, ***P*<0.01 and ****P*<0.001. The values in parenthesis are the LSD at *P*=0.05.

| Source of variability | Grain yield | Filled pod number | Hundred-grain weight | Grain number | WUE | Daily water use |
| --- | --- | --- | --- | --- | --- | --- |
| G | *(1.8) | ***(17.8) | ***(0.7) | ***(18.5) | ***(0.03) | ***(29) |
| W | ***(1.7) | ***(12.6) | **(0.5) | ***(13.1) | ***(0.04) | ***(21) |
| P | ***(1.7) | ***(15.4) | *(0.5) | ***(16.0) | n.s | ***(25) |
| GxW | ***(3.0) | **(25.1) | ***(0.9) | ***(26.1) | n.s | ***(41) |
| GxP | n.s | n.s | ***(1.2) | *(32.0) | n.s | **(51) |
| WxP | ***(2.6) | *(21.8) | *(0.8) | ***(22.6) | **(0.05) | ***(36) |
| GxWxP | ***(5.1) | *(43.5) | ***(1.6) | **(45.2) | ***(0.11) | *(72) |
